# Clinical Characteristics, Treatment Outcomes and Predictive Factors in Optic Neuritis

**DOI:** 10.2174/1874364101812010247

**Published:** 2018-08-31

**Authors:** Linda Hansapinyo, Chayanee Vivattanaseth

**Affiliations:** Department of Ophthalmology, Faculty of Medicine, Chiang Mai University, Chiang Mai, 50200, Thailand

**Keywords:** Optic neuritis, Neuromyelitis optica spectrum disease, Multiple sclerosis, Best corrected visual activity, ONTT, Idiopathic patients

## Abstract

**Background:**

The causes, clinical presentations and treatment outcomes of optic neuritis are distinct among different populations. Early diagnosis based on clinical presentations plays an important role in treating optic neuritis patients.

**Objective:**

The study aimed to determine clinical characteristics, treatment outcomes and predictive factors of treatment outcomes in optic neuritis patients with and without demyelinating disease.

**Methods:**

A retrospective descriptive study of optic neuritis patients carried out between January 2009 and December 2016 was done. Univariate analysis and multivariate logistic regression analysis were used to evaluate the predictive factors of treatment outcomes.

**Results:**

Among 150 patients with optic neuritis, 58 patients were diagnosed with Neuromyelitis Optica Spectrum Disease (NMOSD), 23 patients were diagnosed with Multiple Sclerosis (MS) and 69 patients were idiopathic. The age at presentation in the NMOSD group was significantly younger than the MS group and the idiopathic group. The female:male ratio was significantly lower in the idiopathic group than in the NMOSD group. The initial Best Corrected Visual Activity (BCVA) of 20/20-20/60 (*p* = 0.001) and the idiopathic group (*p* =0.030) was associated with good visual outcomes. Initial BCVA of < 20/200 (*p* = 0.009) and the NMOSD group (*p* < 0.001) was associated with poor visual outcomes.

**Conclusion:**

NMOSD is a more common cause of optic neuritis than MS in Thai population. Female patients with poor initial VA, poor response to steroids treatment, and presenting recurrent attacks are highly suspicious for NMOSD. Optic neuritis without associated demyelinating disease has a better visual outcome and lower recurrence rate.

## 1 INTRODUCTION

Optic neuritis is an inflammatory disease of the optic nerve that typically manifests as an acute visual loss and pain upon eye movements. The causes of optic neuritis could be from demyelinating optic neuritis, which eventually develops into clinically definite Multiple Sclerosis (MS), or from severe immune-mediated demyelinating disease affecting optic nerves as part of Neuromyelitis Optica Spectrum Disease (NMOSD), infection, autoimmune disease, vaccination, and idiopathic [1-4].

Most of the typical form of optic neuritis occurs idiopathically or MS-related. Based on the Optic Neuritis Treatment Trial (ONTT), both idiopathic and MS-related have positive treatment results [5]. In contrast, the majority of atypical optic neuritis has poor visual recovery if left untreated or due to delayed treatment [3, 6-9]. Etiologies of the atypical form of optic neuritis include NMOSD, autoimmune disease, and infection. Many studies have shown differences in gender distribution, a mean age of disease, clinical presentations and post-treatment visual outcomes between Asian and Western population. NMOSD was more common among Asian than Western population, which had poorer visual outcomes [10-17]. Furthermore, only a few studies compared clinical characteristics and treatment results in a different type of optic neuritis in Asians. Early diagnosis based on clinical presentations plays an important role in treating optic neuritis patients. We aim to establish clinical characteristics and treatment outcomes in optic neuritis with and without demyelinating disease (NMOSD, MS), and identify predictive factors of treatment outcomes in Thai population.

## 2 MATERIALS AND METHODS

Electronic medical records of 163 patients who were diagnosed as optic neuritis in neuro-ophthalmology clinic and neurology clinic, Chiang Mai University Hospital between 1st January 2009 and 31st December 2016 were reviewed retrospectively after the Institutional Review Board approval. The criteria for diagnosed optic neuritis were based on ONTT guidelines [2]. We classified patients into three groups, the NMOSD group, the MS group and the idiopathic group. For diagnosis of NMOSD, we followed the international consensus diagnostic criteria for NMOSD with or without Aquaporin-4 Immunoglobulin (AQP4-IgG) status [18]. The diagnosis of MS was based on McDonald criteria 2010 [19]. The exclusion criteria were age lower than 18 years old, and patients with secondary causes of optic neuritis or optic neuropathy such as infection, vaccination, compression, and ischemia. Patients who followed up less than the period of 12 months or incomplete medical data were also excluded from the study.

Demographic data including gender, age of onset, clinical characteristics, affected eye, and optic disc appearance were recorded. The Best Corrected Visual Acuity (BCVA) was examined by Snellen chart and converted into a Logarithm of the Minimal Angle of Resolution (LogMAR) units for statistical analysis. Laboratory tests including complete blood count, antinuclear antibody, rheumatoid factor, human immunodeficiency virus antibody, venereal disease research laboratory test, Treponema pallidum haemagglutination test, toxoplasmosis antibody and AQP4-IgG, chest film, Ishihara color vision test, visual field test (30-2 Humphrey automated perimetry or Goldmann kinetic perimetry), MRI brain and orbit results were also reviewed. The number of recurrent attacks in the affected and fellow eye were recorded. Clinical data at final visit were collected and predictive factors were identified for treatment results. A good visual outcome was determined as final BCVA equal to or better than 20/60, and a poor visual outcome was determined as final BCVA of less than 20/200. For patients with simultaneous bilateral involvement, clinical data of the worse eye would be selected for statistical analysis.

## 3 STATISTICAL ANALYSIS

All statistics were calculated using *SPSS* version 23.0 (*SPSS* Inc., Chicago, Illinois, USA). Continuous data were expressed as mean ± 1 Standard Deviation (SD), and compared using t- test or Mann-Whitney U test as appropriate. Categorical data were expressed in percentages or ratios and compared using the Chi-square test. Univariate analysis and multivariate logistic regression analysis were used to evaluate the predictive factors of treatment results. The *p*-value of 0.05 or less was considered as statistically significant.

## 4 RESULTS

The electronic medical records of 163 patients were reviewed. We excluded 6 patients diagnosed with secondary causes of optic neuritis, such as syphilis, human immunodeficiency virus infection and vaccination. Seven patients followed up for less than 12 months were also excluded. One hundred and fifty patients were recruited into the study. Among 150 patients with optic neuritis, 58 (38.7%) patients were diagnosed with NMOSD, 23 (15.3%) patients were diagnosed with MS and 69 (46.0%) patients were idiopathic. The mean of total medical follow up time +/- SD was 4.40 +/- 2.65 years. The demographic data and clinical manifestations of different groups of optic neuritis are summarized in Table **1**. The mean age of onset ± 1 SD was 39.1 ± 14.5 years (range 18 - 73 years), 45.6 ± 10.2 years (range 19 - 56 years) and 45.4 ± 12.8 years (range 19 - 73 years) in NMOSD, MS, idiopathic groups respectively. The mean age of onset in NMOSD group was significantly younger than the MS group and the idiopathic group (*p* = 0.046 and *p* = 0.007 respectively).

The female:male ratio was significantly lower in the idiopathic group (41:28 or 1.46:1) than in the NMOSD group (57:1, *p* < 0.001) and the MS group (23:0, *p* < 0.001). The frequencies of bilaterality and number of patients with associated autoimmune disease were similar among the three groups. There were two cases of myasthenia gravis and one case of Sjogren syndrome in the NMOSD group. One patient had a history of rheumatoid arthritis in the MS group. One case of systemic lupus erythematosus and another case of autoimmune idiopathic hemolytic anemia were demonstrated in the idiopathic group. The ocular pain was reported at a lower rate in the NMOSD group than the MS group (*p* = 0.031) and the idiopathic group (*p* = 0.023). Patients in the idiopathic group had lower frequencies of normal disc appearance than the NMOSD group (*p* < 0.001) and the MS group (*p* = 0.014). The presence of disc edema and disc hemorrhage in the idiopathic group was statistically more significant than the NMOSD group and the MS group. The number of patients with initial BCVA between 20/20-20/60 was significantly higher in the idiopathic group than the NMOSD group (*p* = 0.001) and the MS group (*p* = 0.036). On the other hand, the number of patients with initial BCVA less than 20/200 was significantly lower in idiopathic group than the NMOSD group (*p* = 0.004) and the MS group (*p* = 0.032).

Ninety-eight percent of patients had abnormal color vision. The most frequent visual field defect pattern is diffuse loss (12.1% in NMOSD, 13.0% in MS, 17.4% in idiopathic). Other visual field defect patterns were demonstrated including central scotoma, cecocentral scotoma, enlarged blind spot, paracentral scotoma, altitudinal defect and hemianopic defect. However, the percentage of patients who had poor initial visual acuity and could not perform visual field testing was high in all groups (39.7% in NMOSD, 30.4% in MS, 23.2% in idiopathic).

One hundred and forty-seven patients received 1000 milligrams intravenous methylprednisolone daily for 3 - 5 days and followed with or without a subsequent tapering dose of oral prednisolone for 2 weeks to 6 months, depending on the etiology of optic neuritis [1, 3, 20]. Three patients in the idiopathic group had spontaneous visual improvement without treatment. Patients diagnosed with NMOSD and MS received long-term immunosuppressants or immunomodulator at neurology clinic. There was no compatible patient with the definition of Chronic Relapsing Inflammatory Optic Neuropathy (CRION) demonstrated in our study [21].

Table **2** compares the treatment results including mean LogMAR VA at final visit and number of recurrent attacks of optic neuritis in different groups. The mean LogMAR VA at the final visit in the NMOSD group was statistically significantly worse than the MS group (*p* < 0.001) and the idiopathic group (*p* < 0.001). The number of patients with final BCVA between 20/20-20/60 was significantly higher in the idiopathic group than the NMOSD group (*p* < 0.001). On the other hand, the number of patients with final BCVA less than 20/200 and less than 10/200 was statistically significantly higher in the NMOSD group than the MS group and the idiopathic group. The proportion of patients with no light perception at the final visit was similar among three groups. Vision improvement after treatment was higher in the MS group (*p* < 0.001) and the idiopathic group (*p* < 0.001) compared with the NMOSD group. Worsening of vision after treatment was higher in the NMOSD group than the idiopathic group (*p* = 0.017). The number of recurrent attacks in the same eye was lower in the idiopathic group than the NMOSD group (*p* < 0.001) and the MS group (*p* < 0.001). The number of recurrent attacks in the fellow eye was higher in the NMOSD group than the idiopathic group (*p* < 0.001).

The predictive factors for treatment results using univariate and multivariate analysis in this study are shown in Tables **3** and **4**. The variables predicting good visual outcomes (BCVA of 20/20-20/60) were the following: age (*p* = 0.009), initial VA of 20/20-20/60, and idiopathic group (*p* < 0.001). On multivariate analysis, initial BCVA of 20/20-20/60 (*p* = 0.001) and the idiopathic group (*p* = 0.030) was independently associated with good visual outcomes. (Table **3**) The variables predicting poor visual outcomes (BCVA of <20/200) were the following: sex (*p* = 0.040), initial BCVA of <20/200 (*p* < 0.001) and NMOSD (*p* < 0.001). On multivariate analysis, initial BCVA of <20/200 (*p* = 0.009) and NMOSD (*p* < 0.001) was independently associated with poor visual outcomes (Table **4**).

## 5 DISCUSSION

In this study, we compared the clinical characteristics and predictive factors of treatment results of optic neuritis in Thai patients. The most common cause of optic neuritis was idiopathic (46.0%). NMOSD was found in 36.7% of patients, which is different from previous studies. In optic neuritis patients, AQP4-IgG was found in 0-11.4% patients in western countries and 9 – 35.2% in Asian countries [9-16, 22, 23]. This rate increased to 20-69.2% in recurrent optic neuritis [10, 13, 24]. We found 57.4% of the patients having at least one recurrent attack. Optic neuritis with MS was found in 23-39.2% in western countries and 9-10% in Asian countries [9, 10, 14-16]. and 15.3% in our study. A recent study examined stored serum available from patients in the ONTT. None of the 177 patients were positive for AQP4-IgG [25]. This implies that NMO is a more common demyelinating disease of the central nervous system than MS for Asian population.

The mean age of the first presentation of optic neuritis in the NMOSD group was 39.1 ±14.5 years (range 18-73 years) which was significantly younger than MS and idiopathic group in our study. These findings are similar to other studies reported by Zhou *et al*. [23]. (mean 34.6 years, range18–55 years). Li *et al*. [24]. (36.53 ± 16.26 years). Although mean age of the first presentation of optic neuritis in NMOSD group was younger than MS and idiopathic group, the oldest patient in the NMOSD group was 75 years old in our study, and there were several studies that reported NMOSD in elderly up to 90 years old [26, 27]. For elderlies with optic neuritis, NMOSD should be taken into account, especially for elderlies with poor vision at the beginning. The female:male ratio was significantly lower in the idiopathic group (1.46:1) than in the NMOSD group (57:1) and the MS group (23:0) in our study. We found only one male patient in the NMOSD group and no male patient in the MS group. These findings are different from other studies; the female:male ratio was 3:1-10.7:1 in the NMOSD group and 1.5:1-5.9:1 in the MS group [28-32], which is lower than the results of our study. Simultaneous bilateral involvement was found in 29.3% in the NMOSD group, 26.1% in the MS group and 21.7% in the idiopathic group which were not statistically significantly different among three groups. Bilateral optic neuritis is common in pediatric patients, Asian and black South African patients [20, 33-36]. Fernandes DB, *et al*. reported 22% of NMOSD group and 37.5% of MS group had bilateral involvement [37]. The frequency of bilateral presentation is affected by age, ethnicity, and causes of disease.

The rate of ocular pain was the lowest in the NMOSD group (19.0%), compared to the MS group (43.5%) and the idiopathic group (37.7%). The ONTT group reported 92% of patients had pain upon eye movement, which is much higher than our patients. We found a high percentage of disc edema (34/69, 49.3%) in the idiopathic group which is similar to Japanese and Taiwanese studies [38, 39], but higher than reported by ONTT group [2]. Disc hemorrhages generally are rare in optic neuritis but are commonly found in anterior ischemic optic neuropathy [40]. Nevertheless, we found 17.2% in the idiopathic group which is higher than the NMOSD group and the MS group. The present study showed the majority of optic neuritis patients associated with demyelinating disease had normal disc appearance (89.6% in NMOSD, 78.3% in MS), which is quite different compared to other studies [2, 24, 41]. The final report by ONTT group about MS risks after optic neuritis concluded that patients without MRI brain lesion at the beginning of isolated optic neuritis attack did not develop MS when baseline exam showed severe optic disc swelling, disc or peripapillary hemorrhages, retinal or macular exudates and absence of pain. We found 56.5% of MS patients who did not complain about ocular pain at the onset of optic neuritis. Since most of the patients recruited in ONTT were Caucasian, the clinical characteristics may differ from Asian and Thai population. Regarding visual acuity on the first presentation of optic neuritis, the idiopathic group had the highest percentage of good BCVA (VA of 20-20/60) and the lowest percentage of poor BCVA (VA of < 20/200) among the three groups. Consistent with the previous studies [6-8, 41], the NMOSD patients had poor initial BCVA.

For the treatment results, the NMOSD patients had the worst final BCVA, the highest rate of VA worsening after treatment and a high percentage of recurrent attacks comparatively among the three groups. On the other hand, the idiopathic group had the best final BCVA, the highest rate of VA improvement after treatment and the lowest percentage of recurrent attacks comparatively among the three groups. Only about 40.5% of our optic neuritis patients achieved final BCVA of 20/20, which is a much lower proportion than reported by ONTT. The majority of patients in ONTT were found to be idiopathic or MS-related who had positive treatment results. In contrast to the present study, NMOSD was found in a higher percentage than MS which had poorer visual outcomes.

Limitations of our study included a retrospective nature, incomplete and varied data documentation. The follow-up interval was different . Further prospective studies to determine the appropriate diagnostic and treatment protocol of optic neuritis in Thailand should be performed.

## CONCLUSION

NMOSD is a more common cause of optic neuritis than MS in Thai population. Female patients with poor initial VA, poor response to steroids treatment, and presenting recurrent attacks are highly suspicious for NMOSD. Optic neuritis without associated demyelinating disease has a better visual outcome and lower recurrence rate.

## Figures and Tables

**Table 1:** Demographic data and clinical characteristics of the different types of optic neuritis.

–	NMOSD	MS	Idiopathic	*p*1 value	*p*2 value	*p*3 value
				NMOSD & MS	NMOSD & Idiopathic	MS & Idiopathic
Number of patients	58	23	69	–	–	–
Mean age at onset ± SD (years)	39.1+/-14.5	45.2+/-10.2	45.4+/-12.8	0.046	0.007	0.96
Female:male (N)	57:1	23:0	41:28	0.84	<0.001	<0.001
Unilateral (N, RE/LE)	41(24/17)	17(12/5)	54(27/27)	–	–	–
Bilateral (N, %)	17 (29.3)	6 (26.1)	15 (21.7)	0.766	0.333	0.681
Ocular pain (N, %)	11 (19.0)	10 (43.5)	26 (37.7)	0.031	0.023	0.599
Normal disc appearance (N, %)	50 (86.2)	18 (78.3)	36 (52.2)	0.461	<0.001	0.014
Abnormal disc appearance (N, %)	8 (13.8)	5 (21.7)	33 (47.8)	–	–	–
- Edema (N, %)	8 (13.8)	5 (21.7)	33 (47.8)	0.462	<0.001	0.01
- Disc hemorrhage (N, %)	0 (0)	1 (4.3)	12 (17.4)	0.517	<0.001	0.048
Initial BCVA	–	–	–	–	–	–
- mean LogMAR visual acuity ± SD	1.88+/-0.84	1.66+/-0.87	1.35+/-0.93	0.309	0.001	0.149
- 20/20-20/60 (N, %)	6 (10.3)	3 (13.7)	23 (33.3)	0.784	0.001	0.036
- 20/70-20/200 (N, %)	9 (15.6)	3 (13.7)	12 (17.4)	0.787	0.777	0.626
- <20/200 (N, %)	43 (74.1)	17 (74.0)	34 (49.3)	0.985	0.004	0.032
Associated autoimmune disease (N, %)	3 (5.1)	1 (4.3)	2 (2.9)	0.866	0.519	0.761

Abbreviations: NMOSD = neuromyelitis optica spectrum disease; MS = Multiple Sclerosis. p1 = NMOSD *versus* MS; p2 = NMOSD *versus* idiopathic; p3 = MS versus idiopathic SD = standard deviation; N. = number; BCVA = best corrected visual acuity. LogMAR = logarithm of the minimal angle of resolution.

**Table 2:** Treatment outcomes of the different types of optic neuritis.

–	NMOSD	MS	Idiopathic	*p*1 value	*p2* value	*p*3 value
				NMOSD&MS	NMOSD&idiopathic	MS&idiopathic
Number of patients	58	23	69	–	–	–
Final BCVA	–	–	–	–	–	–
- mean Log MAR visual acuity ± SD	1.55+-1.02	0,70+-0.81	0.51+-0.74	<0.001	<0.001	0.358
- 20/20-20/60 (N, %)	18 (31.0)	12 (52.2)	47 (68.1)	0.073	<0.001	0.165
- 20/70-20/200 (N, %)	5 (8.6)	5 (21.7)	12 (17.4)	0.134	0.166	0.61
- <20/200 (N, %)	35 (60.3)	6 (26.1)	10 (14.5)	0.001	<0.001	0.262
- <10/200 (N, %)	30 (51.7)	5 (21.7)	6 (8.7)	0.003	<0.001	0.181
- no perception of light (N, %)	6 (10.3)	0 (0)	2 (2.9)	0.062	0.063	0.59
Visual acuity outcome	–	–	–	–	–	–
- better (N, %)	30 (51.7)	18 (78.3)	49 (71.0)	<0.001	<0.001	0.510
- same (N, %)	18 (31.0)	3 (13.0)	13 (18.8)	0.606	0.101	0.489
- worsen (N, %)	10 (17.2)	2 (8.7)	7 (10.1)	0.135	0.017	0.798
Recurrent attacks in affected eye (N of attack)	36 (62.1)	16 (69.6)	16 (23.2)	0.507	<0.001	<0.001
Recurrent attacks in fellow eye (N of attack)	28 (48.3)	7 (30.4)	9 (13.0)	0.094	<0.001	0.095

Abbreviations: NMOSD = Neuromyelitis Optica Spectrum Disease; MS = Multiple Sclerosis. p1 = NMOSD *versus* MS; p2 = NMOSD *versus* idiopathic; p3 = MS *versus* idiopathic. BCVA = Best Corrected Visual Acuity; LogMAR = Logarithm of the Minimal Angle of Resolution. SD = Standard Deviation; N = Number.

**Table 3:** Predictive factors analysis for good visual outcome.

–	Final BCVA ≥20/60 N (%)	Final BCVA<20/60 N (%)	Univariate Analysis		Multivariate Analysis	
Predictive Factors			Odds ratio (95%CI)	*p* value	Odds ratio (95%CI)	*p* value
Mean age at onset (years)	40.25	45.64	1.034 (1.009-1.061)	0.009	1.061(1.025-1.098)	0.068
Female:male (N)	61:16	60:13	0.826 (0.366-1.864)	0.645	–	–
Bilaterality	19 (50)	19 (50)	1.074 (0.514-2.242)	0.849	–	–
Ocular pain	63 (48.1)	68 (51.9)	0.948 (0.857-2.199)	0.744	–	–
Normal disc appearance	51 (49.0)	53 (51.0)	1.431 (0.714-2.859)	0.313	–	–
Abnormal disc appearance	–	–	–	–	–	–
- Edema	26 (56.5)	20 (43.5)	0.740 (0.368-1.488)	0.398	–	–
- Disc hemorrhage	8 (66.67)	4 (33.33)	0.500 (0.144-1.738)	0.275	–	–
Initial BCVA >20/60	27 (84.4)	5 (15.6)	0.136 (0.049-0.378)	<0.001	0.328 (0.089-1.208)	0.024
Cause of optic neuritis	–	–	–	–	–	–
- NMO	18 (31.0)	40 (69.0)	1.959 (1.672-4.006)	0.768	–	–
- MS	12 (25.2)	11 74.8)	0.961 (0.395-2.338)	0.93	–	–
- Idiopathic	47 (68.1)	22 (31.2)	0.275 (0.140-0.542)	<0.001	2.803 (0.977-8.044)	0.030

Abbreviations: BCVA = Best Corrected Visual Acuity; N = Number; NMOSD = Neuromyelitis Optica Spectrum Disease; MS = Multiple Sclerosis;

**Table 4:** Predictive factor analysis for poor visual outcome.

–	Final BCVA<20/200 N (%)	Final BCVA≥20/200 N (%)	Univariate analysis		Multivariate analysis	
Predictive factors			Odds ratio (95%CI)	*p* value	Odds ratio (95%CI)	*p* value
Mean age at onset (years)	45.19	41.87	0.981 (0.956-1.007)	0.153	–	–
Female:male (N)	45:5	76:24	2.944 (1.05-8.255)	0.040	0.714 (0.171-2.981)	0.644
Bilaterality	18 (47.7)	20 (52.3)	0.464 (0.218-0.988)	0.040	0.435 (0.171-1.111)	0.082
Ocular pain	17 (37.0)	29 (63.0)	0.829 (0.401-1.712	0.611	–	–
Normal disc apperance	38 (36.5)	66 (63.5)	0.757 (0.360-1.592)	0.463	–	–
Abnormal disc appearance	–	–	–	–	–	–
- Edema	13 (28.2)	33 (71.7)	1.321 (0.628-2.779)	0.463	–	–
- Disc hemorrhage	4 (33.3)	8 (66.7)	1.175 (0.344-4.018)	0.797	–	–
Initial BCVA <20/200	44 (46.8)	50 (53.2)	0.162 (0.067-0.395)	0.000	0.178(0.068-0.462)	0.009
Cause of optic neuritis	–	–	–	–	–	–
- NMO	35 (60.3)	23 (39.7))	0.138 (0.065-0.294)	0.000	0.148 (0.067-0.330)	0.001
- MS	6 (26.1)	17 (73.9)	1.555 (0.572-4.223)	0.387	–	–
- Idiopathic	10 (14.5)	59 (85.5)	6.047 (2.716-13.450)	0.879	–	–

Abbreviations: BCVA = Best Corrected Visual Acuity; N = Number. NMOSD = Neuromyelitis Optica Spectrum Disease; MS = Multiple Sclerosis.
